# Anorexia Nervosa and a Lost Emotional Self: A Psychological Formulation of the Development, Maintenance, and Treatment of Anorexia Nervosa

**DOI:** 10.3389/fpsyg.2019.00219

**Published:** 2019-03-04

**Authors:** Anna Oldershaw, Helen Startup, Tony Lavender

**Affiliations:** ^1^Salmons Centre for Applied Psychology, Canterbury Christ Church University, Canterbury, United Kingdom; ^2^Kent and Medway All Age Eating Disorder Service, North East London NHS Foundation Trust, London, United Kingdom; ^3^Sussex Eating Disorders Service and Research and Development Department, Sussex Partnership NHS Foundation Trust, Sussex, United Kingdom

**Keywords:** Anorexia Nervosa, emotion regulation, emotion, eating disorders, psychological therapy

## Abstract

In this paper, we argue that Anorexia Nervosa (AN) can be explained as arising from a ‘lost sense of emotional self.’ We begin by briefly reviewing evidence accumulated to date supporting the consensus that a complex range of genetic, biological, psychological, and socio-environmental risk and maintenance factors contribute to the development and maintenance of AN. We consider how current interventions seek to tackle these factors in psychotherapy and potential limitations. We then propose our theory that many risk and maintenance factors may be unified by an underpinning explanation of emotional processing difficulties leading to a lost sense of ‘emotional self.’ Further, we discuss how, once established, AN becomes ‘self-perpetuating’ and the ‘lost sense of emotional self’ relentlessly deepens. We outline these arguments in detail, drawing on empirical and neuroscientific data, before discussing the implications of this model for understanding AN and informing clinical intervention. We argue that experiential models of therapy (e.g., emotion-focused therapy; schema therapy) be employed to achieve emergence and integration of an ‘emotional self’ which can be flexibly and adaptively used to direct an individual’s needs and relationships. Furthermore, we assert that this should be a primary goal of therapy for adults with established AN.

## Introduction

Anorexia Nervosa (AN) is an eating disorder (ED) characterized by self-starvation driven by weight, shape and eating concerns and extreme dread of food, eating and normal body weight (American Psychological Association [APA], 2013; Walsh, 2013; Treasure et al., 2015b). The annual United Kingdom female incidence of AN is approximately 14 cases per 100,000 (Micali et al., 2013), with up to 4% of women and 0.24% of men meeting the broad definition of AN in their lifetime (Smink et al., 2013). The peak age of onset for girls is 15–25 years and for boys is 10–14 years (Micali et al., 2013). AN is associated with poor prognosis and the highest mortality rates of all psychiatric disorders (Smink et al., 2013).

The treatment of choice for AN is talking therapy (National Institute for Health and Care Excellence [NICE], 2017). Yet the disorder has poor rates of remission and high levels of relapse. Current psychological interventions facilitate small change, with better interventions needed (Bulik, 2014; Startup et al., 2015). Although early intervention is key to recovery, there is an average delay of 18 months from symptoms emerging to treatment, followed by multiple relapses even following treatment, each lasting 6 years (PricewaterhouseCoopers, 2015). Costs of AN and other EDs to the individual, family and carers, and society are therefore substantial. Time spent caregiving for somebody with severe AN is almost twice that for somebody with a physical health disorder (e.g., cancer) or other mental health difficulty (e.g., psychosis; (Viana et al., 2013). The annual cost to the United Kingdom economy is estimated to be £17.9 billion, offering a “compelling case for change” in services and treatment (PricewaterhouseCoopers, 2015, p. 9). There is now significant work underway in this vein; for example, the First Episode and Rapid Early Intervention for Eating Disorders (FREED; Brown et al., 2018).

### Risk and Maintenance Factors Associated With AN

**FIGURE 1:**
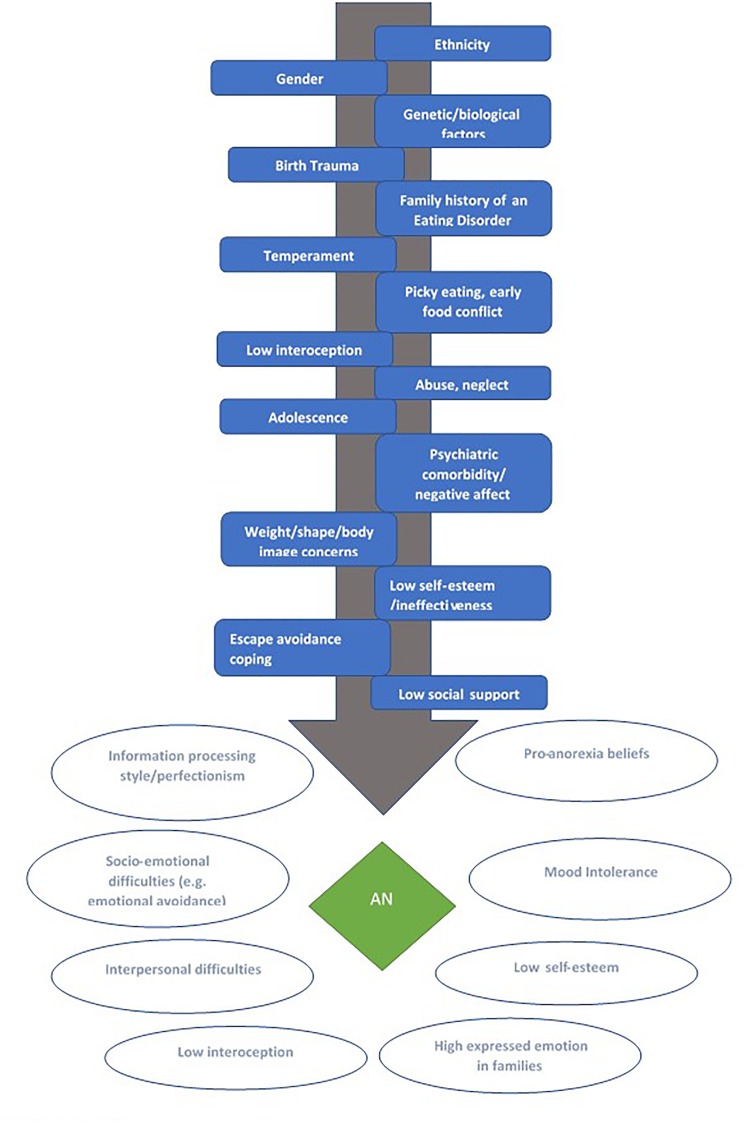
Summary of risk and maintenance factors for Anorexia Nervosa, adapted form several sources (Stice, 2002; Jacobi et al., 2004; Striegel-Moore and Bulik, 2007; Treasure and Schmidt, 2013). Putative risk factors are depicted 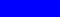 and maintenance factors depicted 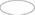.

Anorexia Nervosa has been associated with numerous broad ranging risk and maintenance factors. *Risk factors* are variables which predict subsequent development of later pathology, in an individual currently disorder and symptom-free (Stice, 2002). Risk factors can have effects mitigated by *protective factors* or amplified by *potentiating factors*. Maintenance factors predict symptom persistence versus remission over time in individuals already symptomatic for a disorder (Stice, 2002). Clinical implications of risk and maintenance factors differ; risk factors are relevant to the development of preventative programs and maintenance factors to treatment interventions (Stice, 2002). While we do not fully review evidence for risk and maintenance factors for AN herein; we briefly indicate those most well-accepted and summarize them diagrammatically in Figure 1. This reveals complex interactions between genetic, biological, psychological, and socio-environmental factors in the development and maintenance of AN, with some factors proposed to represent both risk and maintenance factors.

### Current Clinical Perspectives, Models, and Treatment for AN to Date

Psychological models to date use existing psychological theory to address relevant risk or maintaining factors within clinical treatment for AN. Recent guidance published by National Institute for Health and Care Excellence [NICE] (2017) recommends the following psychological interventions be considered for adults with AN: Eating Disorder Focused Cognitive-Behavioral Therapy (CBT-ED); Maudsley Anorexia Nervosa Treatment for Adults (MANTRA); Specialist Supportive Clinical Management (SSCM); and Focal Psychodynamic Therapy (FPT).

CBT-ED traditionally focuses on symptom-based accounts suggesting both control and overvaluation of weight/shape maintain AN. Later revisions include: clinical perfectionism; low self-esteem; mood intolerance; and, interpersonal difficulties as additional treatment foci (Fairburn et al., 1999, 2003, 2009, 2015). Using cognitive and behavioral techniques, it seeks to increase motivation to change, directly enhance weight gain while tackling concerns about weight and shape and prepare for set-backs to maintain gains made (Fairburn et al., 2009).

MANTRA outlines a broader range of putative maintenance factors as treatment targets (Schmidt and Treasure, 2006; Wade et al., 2011; Schmidt et al., 2012, 2015; Treasure and Schmidt, 2013). Four core factors are included: (1) Rigid, detail-focused, and perfectionist information processing style; (2) Socioemotional difficulties (e.g., avoiding experience and expression of emotions within close relationships); (3) Positive beliefs about the value of AN; and (4) Close others exhibiting high expressed emotion or accommodating/enabling AN behaviors. These factors intensify once in a starved state, further maintaining them. The authors argue that CBT models miss important factors because key difficulties underlying EDs rarely concern ED-related themes (only 1%); with issues of interpersonal difficulties being more significant, including rejection and abandonment (42%); negative self-perception (22%); and emotional experience (20%; Sternheim et al., 2012).

Focal Psychodynamic Therapy employs three phases of treatment focussing upon relationships and breaking pro-anorexic belief and behavior patterns (Zipfel et al., 2014). Firstly, it concentrates upon relationship building, therapeutic alliance, identifying pro-anorectic behavior/beliefs and self-esteem. Secondly, relevant relationships are examined and links with AN beliefs/behaviors made. Finally, this is transferred to everyday life and a therapeutic ending.

Specialist SSCM (McIntosh et al., 2006) was originally developed as a comparison ‘treatment as usual’ for use in clinical trials. It combines clinical management and supportive psychotherapy to provide practical support and is not formulation-based; rather it focuses on psycho-education, resumption of eating and normalization of weight.

These treatment developments since the publication of previous NICE guidelines (National Institute for Health, and Care Excellence [NICE], 2004) represent obvious innovation in the application of empirical research to treatment models and clinical practice. However, of these, there remains no clear front runner and it is uncertain which treatment best suits which sufferer of AN. Results from randomized controlled trials (RCTs) indicate that these speciality out-patient treatments do not out-perform each other or control comparisons post-therapy or at follow-up (Carter et al., 2011; Schmidt et al., 2012, 2015; Zipfel et al., 2014; Fairburn et al., 2015). They demonstrate only small non-significant effect sizes of change (Watson and Bulik, 2013), with around 20% of people weight-restored after 1 year (Schmidt et al., 2012, 2015; Zipfel et al., 2014). Therefore, non-specific control interventions seeking to manage clinical symptoms (e.g., SSCM) appear as effective as complex, empirically driven models, prompting the inclusion of SSCM in NICE guidelines. This falls short of advances made in outcomes from interventions developed for other Axis I disorders, including CBT for depression, generalized anxiety disorder, panic disorder, obsessive compulsive disorder, and Bulimia Nervosa (Butler et al., 2006).

Anorexia Nervosa is notoriously considered ‘difficult to treat’ and, as described, treatment outcomes indicate an unexplained discrepancy between theoretical models based on empirical data findings and clinical application. It may be that even where causal models are available and appear robust, it cannot be assumed that derived interventions effectively manipulate targets. Evidence of the impact of interventions upon proposed maintenance factors is absent in the field and not understanding how change is facilitated is a barrier to developing evidence-based interventions for AN (Pennesi and Wade, 2016). Furthermore, speciality interventions developed to date tend to have complex hypotheses with many diverse target variables. This potentially falls into the trap of an unhelpful ‘everything is relevant’ approach common in mental health research and results in the inclusion of many possible risk or maintenance factors into a causal model (Kendler and Campbell, 2009). It produces heterogeneity across the delivery of an intervention creating difficulty in drawing links between outcomes and causal processes. In addition, while earlier models have sought to describe and address the current clinical presentation of AN, it is imperative that models offer strong theoretical bases and robust consideration of how AN has arisen; a paradigm that the ED field has not optimally employed (Pennesi and Wade, 2016). Previous models may fail to sufficiently consider and adequately account for etiology and phenomenology of EDs (Fox, 2009; Waller et al., 2010). Like others, we therefore propose that any explanation for AN must include reference to phenomenological and interpretive aspects of the presentation (Amianto et al., 2016). Moreover, the definition and extrication of risk and maintenance factors is complex, especially for AN which is compounded due to its mixed psychological and physical presentation, and there is a paucity of research examining risk and maintenance factors by differential ED diagnoses (cf. Stice, 2002; Jacobi et al., 2004; Jacobi et al., 2011). Risk factors can be potentiated once in the ill state, or mediated by other variables and maintenance factors can be generated as a consequence of AN, perpetuating the disorder (Wonderlich et al., 2005). Thus, key foci for clinical interventions are difficult to discern and may alter as the disorder progresses.

Building an explanatory developmental framework attempting to understand how factors link together to cause and maintain AN makes it unnecessary to distinguish between risk and maintenance factors and is therefore desirable. We propose that an integrative account of the emergence of risk and maintenance factors and their interplay (including how this is potentiated once AN is established) is required to gain the necessary depth of understanding of the development and presentation of AN to develop and inform interventions.

### Aims of the Current Paper

The current paper offers an account of AN integrating risk and maintenance factors by proposing their influence upon development and course of AN may be explained and potentiated by an underpinning unifying explanatory variable of emotional difficulties, giving rise to AN as a disorder of a ‘lost sense of emotional self.’

First, we describe the difficulties with emotion experienced by people with AN forming the basis of our argument of AN as a ‘lost sense of emotional self’ (see the section “Emotional Difficulties in AN”). We present how emotional difficulties link several known risk and maintenance factors for AN throughout development (see the section “Development of AN”). We describe how, once developed, AN becomes self-perpetuating within this context adding to persistence and complexity of disorder and treatment (see the section “Perpetuation of AN: The Ever Decreasing ‘Self”’). Once this core presentation to treatment is outlined (a ‘lost sense of emotional self’), we offer considerations for clinical conceptualization and practice and hypothesis testing (see the sections “Clinical Conceptualization and Practice” and “Future Directions in Testing the ‘Lost Emotional Self’ Hypothesis”).

## Emotional Difficulties in AN

Emotions evolved as adaptive processes: they are learnt or instinctive responses to external or internal stimuli, informing about immediate environments, relationships and our needs, with critical impact upon physiology, behavior and cognition, including memory and decision-making capabilities (Dolan, 2002). They evolved to organize and direct human cognitions and behavior (Cosmides and Tooby, 2000). Emotions act as a super-ordinate system; we describe them as the conductor of an orchestra comprised of cognitive, behavioral, physiological and social functions (Oldershaw et al., 2015).

Dysfunctional emotional processing and regulation are proposed to underpin many psychological disorders (Aldao et al., 2010), including EDs (Aldao et al., 2010; Hatch et al., 2010b; Arcelus et al., 2013; Lavender et al., 2015; Oldershaw et al., 2015; Mallorqui-Bague et al., 2018). They play a significant role in the development and maintenance of AN (Schmidt and Treasure, 2006; Haynos and Fruzzetti, 2011; Treasure and Schmidt, 2013; Wildes et al., 2014; Racine and Wildes, 2015). Indeed, even earliest descriptions recognize emotional experience as a factor in AN development and maintenance. In 1871, a women with AN was reported to *“suffer from some emotions she avows or conceals”* (Charles Lasègue cited in Vandereycken and Van Deth, 1990). A century later, women with AN were described as having underlying deficiencies in the identification of emotional states and responses (Bruch, 1962). A link between emotional experience and the behavioral expression of AN is clear: potential emotion is avoided by eliciting predictable and controllable behavioral patterns from others (Treasure et al., 2016); focus on food, eating, weight, and shape, as well as cognitive processes such as worry and rumination, affords cognitive distraction from negative thoughts/emotion (Sternheim et al., 2011; Startup et al., 2013). Once starved, suppressed physiological experience numbs emotions and is valued (Miller et al., 2003, 2009; Serpell et al., 2004); while starvation and emaciation enables a maladaptive expression of distress (Serpell et al., 2004) and ever-narrowing interpersonal life fuels greater reliance on AN (Schmidt and Treasure, 2006).

Systematic reviews of emotional experience in AN expand on this understanding by providing summaries of experimental and self- report data (Oldershaw et al., 2011, 2015; Lavender et al., 2015), integrating pre-existing theory from the field of emotion regulation (Aldao et al., 2010; Gross, 2013). The reviews indicate that, relative to healthy controls, people with AN experience difficulties in emotional awareness (including alexithymia and poor emotional clarity), alongside high self-reported levels of disgust and shame specifically. Difficulties in meta-level processes are also evident, such as elevated maladaptive schema, particularly around defectiveness, dependence/incompetence, social isolation and subjugation, and negative beliefs about having or expressing emotion.

**FIGURE 2:**
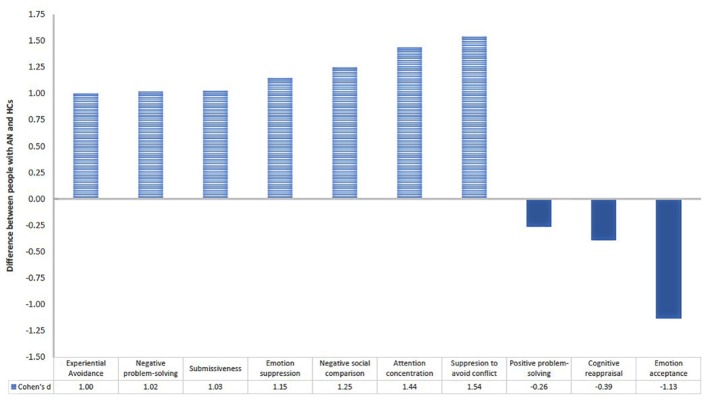
Graph indicating difference between people with Anorexia Nervosa (AN) and healthy comparisons (HCs) in their use of maladaptive 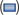 and adaptive 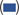 emotion regulation strategies [adapted from Oldershaw et al. (2015)].

Engagement in many emotion regulation strategies is evident, indicative of a pattern of emotion over-regulation (Oldershaw et al., 2015) (Figure 2). Adaptive emotion regulation strategies are absent, and unhelpful strategies dominant, including: avoiding emotion triggers, such as situations or modifying social interaction (e.g., submissiveness); worry and rumination processes; and emotion suppression, particularly to avoid conflict. It is argued that people with AN are disproportionately reliant upon the feedback of others for reassurance and regulation of emotion (e.g., via social comparison). These findings support the notion that emotional avoidance and unhelpful over-regulation strategies play a central role in AN, including those achieved via anorexic behaviors (Dolhanty and Greenberg, 2009; Wildes et al., 2010; Brockmeyer et al., 2012; Arcelus et al., 2013; Treasure and Schmidt, 2013). Indeed, emotional avoidance and submissive behaviors (and not social cognition or neurocognition) are more promising predictors of clinical outcomes following treatment (Oldershaw et al., 2018).

### Emotional Avoidance as a Maintenance Factor for AN

In summary, following many other authors highlighting the key role of emotional avoidance in AN (e.g., Wildes et al., 2010, 2014; Treasure and Schmidt, 2013), experimental and self-report findings point to a maintenance model of AN as a disorder underpinned by difficulties with emotional experience promoting emotional avoidance and over-regulation (Oldershaw et al., 2015). This model posits that early life factors develop schemata and beliefs that leave somebody vulnerable to experiencing emotion as overwhelming and confusing. Emotion regulation strategies, including ED behaviors, develop in this context as a means to control and prevent triggering emotion. Strategies developed are perceived as useful in the first instance, generating an initial reduction in emotion. Ultimately, however, they are maladaptive methods of controlling and regulating emotion serving only to trigger further negative emotional experience and reinforce negative beliefs and schemata, thereby increasing reliance upon (maladaptive) emotion regulation strategies: hence a vicious cycle develops (Oldershaw et al., 2015).

### Emotional Avoidance and the Impact on Development of Self in AN

In the current paper, we consider the implications of this persistent emotional avoidance cycle and seek to further this as an explanation by considering both how it arises and its consequences. We will argue that this picture of emotional avoidance and over-regulation is shaped by and further impacts development of self. As such, we will propose that it is not simply a difficulty with emotional experience, interpersonal experience or emotion regulation that is observed and should be tackled in treatment, but the consequential impact of this on development and awareness of a core ‘emotional self.’

We posit that difficulties with emotion are so pervasive due to their integral part of our existence: the basis of the self and emotion are shared and inextricably linked (Damasio, 2003). Primary emotional experience emerges from physical arousal, including interoceptive awareness, in addition to complex processes such as neural activation (Barrett et al., 2007) and emotional memory networks (Greenberg, 2004). Interoceptive emotion signals afford us a mental representation of selfhood experienced within the body – “a material me” (Seth, 2013). Through such processes, emotion becomes fundamental to the construction and organization of ‘self’ with largely bottom-up interoceptive hierarchies of emotional experience interacting to motivate and direct behavior (Damasio, 2003; Greenberg, 2004). Thus, there is a process of non-conscious emotional experience, giving rise to conscious thinking and feeling states, which are self-regulated (Hatch et al., 2010b).

‘Self’ is the overarching concept used to describe the organization and integration of our many identities; who I am within each relationship and every social interaction (Haworth-Hoeppner and Manies, 2005). It is argued that our identity is ‘rooted in emotion, emerging in relationships, developing as a dynamic self-organizing system’ (Bosma and Kunnen, 2001, p. 5). If we cannot access those bottom-up emotional hierarchies (“a material me”), we are without the conductor of the orchestra (our emotional sense of self). If the conductor is not functioning or appears absent, the orchestra descends into disorder. It must muddle through as best it can, desperate to conceal in-fighting, misattunement and confusion, while persistently reliant upon and sensitive to audience feedback to ascertain if it is doing an adequate job.

People with AN lack interoceptive perception of their own body and the internal bodily sensations which give rise to the basic form of self-awareness and emotion perception (Gaudio et al., 2014; Stanghellini et al., 2015). It is suggested that AN emerges in this context of vague and overwhelming emotional experience both to regulate emotion (Oldershaw et al., 2015) and to regain a sense of bodily self, defined by the other’s gaze and external appearance (Monteleone et al., 2017). Indeed, shape and weight concerns are thought to arise as the result of a disturbance in experience of one’s own body (embodiment) and are influential in how personal identity is determined (Stanghellini et al., 2012). People with AN have described the disorder as a means of forging a new identity (Nordbo et al., 2006) which becomes inexplicably linked with, or replaces, the ‘true’ self (Williams et al., 2016). As such, AN has been referred to as a “false self” (Bruch, 1988), arising due to an otherwise unstable and fragile self (Karwautz et al., 2001b) unable to integrate the body (Amianto et al., 2016). In this paper, we further define this difficulty with self by arguing that AN arises specifically from a *lost emotional* *sense of self*. That is, a person struggling to navigate the world, themselves and others, without an emotional conductor to guide them, increasingly reliant upon feedback of others.

## Development of AN

Here, we build a picture of the development of AN, moving from infancy through to adolescence, seeking to offer an explanatory developmental framework for the emergence of AN in the context of a ‘lost emotional self,’ drawing on identified risk and maintenance factors, and highlighting how they are unified by being both influential upon and potentiated by emotional difficulties.

### Infancy and Early Life

#### Attunement and Attachment

Difficulties with emotional awareness and regulation in EDs are hypothesized to have their origins in childhood attachment (Arcelus et al., 2013). At the earliest stage of infancy, maternal reflective functioning and attunement are critical to emotional development. Reflective functioning is caregiver capacity to hold her child’s emotional and mental states in mind. Attunement is caregiver responsiveness and ability to identify, model and name emotional experiences for a child. They are core to attachment, which enables the capacity to identify the feelings of self and others and to integrate them into a felt emotional experience, forming a template for self and others in close relationships (‘internal working models’; Bowlby, 1969). The mother acts as container for her child’s unbearable feelings, such as anger and distress, with her responses shaping the child’s future responses to emotions (Bion, 1962). Through this process, emerging emotional awareness enables a gradual articulation of self-experience (de Groot and Rodin, 1994) and differentiation (Mahler, 1963). This requires a “goodness-of-fit” between maternal and infant temperament (Belsky, 1984). When present, adequate mutual regulation of interactions and communication of needs and attention are possible. Yet this is an extremely sensitive process; even incongruence as early as 8 months old can impair neural discrimination of emotional facial expressions (Rajhans et al., 2016).

Over 35 years ago, Bruch advocated a role of attachment and early social experiences in the development of AN (Treasure and Cardi, 2017). Insecure patterns of attachment of people with AN to primary caregivers have been reported in adults (Zachrisson and Skarderud, 2010; Tasca and Balfour, 2014) and adolescents (Gander et al., 2015; Jewell et al., 2016; Pace et al., 2016), of large effect (Caglar-Nazali et al., 2014). Further, people with AN have particularly low levels of reflective functioning (Tasca and Balfour, 2014). This matches a similar pattern of attachment in the mothers of those with AN (Ward et al., 2001; Pace et al., 2015). Higher attachment anxiety is significantly related to greater ED severity and poorer treatment outcomes (Illing et al., 2010). A discrepancy in the fit between environment and the temperament of the individual who develops AN is proposed to lead to an experience of social environments as invalidating or demanding (Karwautz et al., 2001a,b; Corstorphine et al., 2007; Oldershaw et al., 2015). Chronic misattunement (“empathic failure”) is argued to affect the development of any or all sense of self and relate to ED development (de Groot and Rodin, 1994).

#### Gender

AN is more prevalent for females than for males with ratio of 1:8 (Steinhausen and Jensen, 2015). Female preponderance of EDs is often hypothesized to reflect cultural ideals, particularly internalization of the thin ideal and objectification of women. Yet this fails to adequately address the complexity of gender and gender role socialization. Gender differences in emotion expression arise as a complex combination of biological determinants, socialization, social context and cultural expectations (Chaplin, 2015). We argue that gender socialization and social construction of emotion expectations from infancy plays a role in women becoming particularly at risk of developing an ED.

From an early age, parents socialize emotional expression based on gender stereotypes or expectations; encouraging internalizing emotions such as sadness, fear, shame and guilt and discouraging anger expression in girls, while the opposite is observed for boys (Zeman et al., 2012). Girls are expected to display a greater array of emotions than boys, including positive emotions (Brody and Hall, 2008), yet, perhaps due to biological differences, need more encouragement to learn to express their emotion than boys (Chaplin, 2015). Consequently, girls have been observed to display more internalizing emotions than boys, with the strongest differences found for fear and shame (Chaplin and Aldao, 2013). High levels of internalizing difficulties and emotions (particularly shame) are prevalent for people with AN (Oldershaw et al., 2015).

The ‘self-in-relation’ (capacity for identification with and relatedness to the mother) is considered more important to female identity than male (Gilligan, 1982). Miscoordinated interactions with the caregiver leads to a sense of ineffectiveness more prone with mother–daughter than with mother–son dyads (Tronick and Cohn, 1989). Mothers see daughters as a continuation of themselves more than they do sons, thus affecting the extent to which girls are experienced as separate and individual (de Groot and Rodin, 1994). Further, girls in particular may base their emotional relatedness on parental feelings rather than their own emotion; facilitating intimacy with the mother, but interfering with girls’ experience of their own unique emotional world (de Groot and Rodin, 1994). Indeed, women can find it difficult to define themselves without first contextualizing ‘self’ within the mother–daughter relationship (Miller-Day, 2012). In general, girls feel less powerful in parental relationships than boys (Buhl, 2008). Thus, females value interconnected social and emotional experience more than males, but at the expense of developing their own unique emotional self-awareness and experience. Such patterns are observed in higher levels with people with AN. They report lower perceived individual autonomy and higher perceived cohesion in their relationship with their mother than unaffected sisters, although have similar perceived emotional connectedness (Karwautz et al., 2003).

Females have greater innate empathy than males, linked not only to socialization, but resulting from evolutionary processes creating neurobiological differences (Christov-Moore et al., 2014). Girls are expected to offer more empathy and sympathy than boys, and are socialized to this end (Knafo et al., 2008). Girls are more sensitive than boys to the responses of others, including both approval and rejection, with girls’ need for approval superseding their striving for autonomy (McClure, 2000). As such, females may consider their own subjective experience or evaluations as less valid than those of others. Indeed, it has been argued that mothers (consciously and unconsciously) direct their daughters in “gender appropriate ways” including offering the message their own “needs” and certain emotions are unacceptable (Orbach, 1993 cited in Richmond, 2002). This can include modeling for girls “feminine” emotion expression such as appearing cheerful, even where this is not felt (Chaplin, 2015). Girls demonstrate greater suppression of emotions, particularly in relation to their own goals or needs (e.g., disappointment; Saarni, 1984).

This presents an image of girls as sensitive to the feelings of others, likely to suppress their own emotions and needs, and to experience the self via the other (mother). These features may be heightened for people who go on to develop AN. People with AN report low levels of their own emotional awareness, alexithymia (inability to identify and describe their own feelings), limited expression of their own emotion and submissiveness, especially to avoid conflict (Oldershaw et al., 2015). They suppress facial expression of emotion and use fewer words when describing their emotional experiences as compared with healthy people or those recovered from AN (Davies et al., 2011, 2012, 2013). They thus inhibit the expression of their own emotion (Gramaglia et al., 2016) and autonomy (Karwautz et al., 2003) while seeming able to acknowledge others’ emotions. Individuals with AN during both starvation and weight restoration report greater empathy than healthy controls (Hambrook et al., 2008), including in the domain of personal distress (vicarious negative arousal to others’ suffering; Beadle et al., 2013). They appear highly motivated to understand the feelings of others, ‘hyperscanning’ stimuli relating to others while avoiding visually attending to salient features of their own facial image (Phillipou et al., 2015). They have low self-directedness, scoring highly on ‘Other-Directedness’ (Bachner-Melman et al., 2009). People currently with AN have significantly less self-focussed attention than those who are weight restored (Zucker et al., 2015), while tending to self-attribute negative over positive social interactions (McAdams et al., 2018). Perhaps unsurprisingly then, adolescents with disordered eating devalue personal subjective experience, and favor socially accepted externally validated ideals (e.g., “thin ideal”) as their own (Steiner-Adair, 1990, cited in de Groot and Rodin, 1994).

It is of note that the male incidence of AN has increased in recent decades, in the absence of any such increase in bulimia nervosa (Steinhausen and Jensen, 2015). Attitudes toward gender roles have shifted substantially since the 1980s, including toward roles in relationships, parenting, and women’s participation in the labor market (Park et al., 2013). It is possible that changes in gender role norms, as well as in emotion socialization may have ensued, including changes in mother–son dyad relationships; further research is required to investigate this. We propose that any such shifts could in part relate to this increase in male AN presentations.

#### Food and Communication

Effectively feeding one’s children is of innate importance, essential for well-being and survival. Up to 52% of infants and toddlers are viewed by caregivers as experiencing feeding problems (Reau et al., 1996). Even feeding healthy children can be time-consuming, tedious and exasperating: up to 15 exposures of a new food can be required before it is trusted enough to even be tasted (Wardle et al., 2005). Moreover, around age two, children undergo a developmental change, causing them to reject foods previously liked and accepted (Cooke et al., 2003) triggering potential frustration and anxiety for parents. Emotions and mealtimes are strongly linked, and although largely positive in valence, negative emotions such as disgust and guilt are aroused in subgroups described as “indifferent restrictives” (den Uijl et al., 2014). Indeed, adults’ food preferences and emotions can be traced back to early negative experiences such as pressure to eat (Batsell et al., 2002), with less pressure predicting food enjoyment (Webber et al., 2010). Moreover, EDs have been retrospectively linked to greater use of food for communication and emotion regulatory purposes, such as rewarding, comforting, or punishing (Mazzeo and Bulik, 2009). Thus, even preverbally, children learn to associate food with feeling misunderstood, stress and anxiety. Further, using food and eating as communication and for emotion regulation may be fostered and is observed in girls as young as five (Carper et al., 2000).

People who develop AN have increased early life experiences generating sensitivity to and preoccupation with somatic experience, including early gastrointestinal events and eating difficulties, with evidence pointing to digestive problems as a risk factor for AN (Marchi and Cohen, 1990; Jacobi et al., 2004). Once people develop AN and caregivers become concerned, food offers a way to communicate needs valued by the person with AN (Serpell et al., 2004). Emotion expressed around eating, and engagement with the ED by parents, may represent an emotional engagement that is reinforcing, but ultimately leaves the sufferer feeling overwhelmed and misunderstood in the moment serving only to remove them further from developing an awareness and acceptance of authentic internal experiences.

#### Infancy and Early Life: Summary

People who develop AN may be those with poor fit in their environment, leading to poor attunement and insecure attachment. Consequently they may feel invalidated as a separate, unique person, and be left with a sense that their own emotions and needs are less important than those of others and should be suppressed. It follows that this could result in poor emotional awareness and regulation, in the context of attempts at pleasing or meeting the emotional needs of others. Even from an early age, food and mealtimes may represent periods of heightened emotion and emotional communication for both parent and child.

### Adolescence

Anorexia Nervosa can affect people of any age, gender, culture; however, adolescent and young adult females are most at risk (Zipfel et al., 2014). AN typically emerges in early- to mid-adolescence (Herpertz-Dahlmann, 2009), highlighting adolescence as a risk factor in its development. Adolescence is a challenging period emotionally and socially with consequences for emotion regulation and psychological adjustment (Miller-Slough and Dunsmore, 2016). It is a critical period for developing identity (Pfeifer and Berkman, 2018) associated with emergence of self-concept and enhanced self-awareness (Weil et al., 2013). The ‘self’ develops driven by a key intersection of social, cognitive, affective, motivational, and regulatory processes (Pfeifer et al., 2013). As such, adolescence is a phase of life representing an opportunity, yet also a vulnerability (Fuhrmann et al., 2015).

#### Parents and Identity

Identity development during adolescence has roots in the parent–child relationship. Separation–individuation factors such as autonomy-supportive parenting, separateness from parents, and personal autonomy are crucial (Luyckx et al., 2006, 2007; Beyers and Goossens, 2008). Early developmental interactions with parents, and whether they met identity needs of parent or child, can have implications for identity development and separation–individuation in adolescence (Koepke and Denissen, 2012). Autonomy supporting parents promote a belief in personal agency by enabling expression of identity and opinion without fear of parental rejection or engulfment (Luyckx et al., 2007). To fully engage in adult-to-adult relationships with grown up children, parents need to let go of the part of their own identity of “omnipresent caretaker” (Koepke and Denissen, 2012). And yet, emotion socialization continues to rely on parents. The need to offer appropriate emotional guidance is enhanced by new social demands and risks (Garcia and Scherf, 2015). Thus, adolescence is a challenging time of adaptation for parents, alongside their child.

Separation–individuation processes during adolescence may be particularly problematic for people who develop AN, and may follow from interactions during early life described above. Women with AN identify boundary violations by their parents within the family (Rowa et al., 2001). Adolescents with AN rate their families as less communicative, flexible, cohesive, and more disengaged, compared to control participants (Laghi et al., 2017). Maternal criticism and emotional over-involvement links to ED severity (Kyriacou et al., 2008; Duclos et al., 2014). High levels of expressed emotion is included in maintenance models of AN and reflects a description of families as critical, hostile and/or emotionally over involved and overprotective, particularly toward the person with AN (Schmidt and Treasure, 2006; Treasure and Schmidt, 2013). Thus, there is a traditional view that families of people with AN are enmeshed and rigid in their style (Minuchin et al., 1978). This fits with the presentation described: a person with AN unsure of their own internal world, seeking to meet the needs of others over their own. Indeed, adolescents with EDs have lower levels of self-differentiation, indicating high emotional reactivity, emotional cut-off, and greater fusion with others, causing confusion between one’s own emotional and mental states and those of others (Doba et al., 2018). Consequently, people with AN report a longing for independence seen as a reaction to helplessness (Karwautz et al., 2001b). Taking such an oppositional stance by refusing to eat in the face of desperate parental persuasion may reflect a need for adversarial transference (de Groot and Rodin, 1994), thus, enabling growth of the person with AN and a sense of separateness through assertion of opposition. The instinct for individuation may also be a factor in seeking external validity of their worth via social reinforcement from others.

Although, some of these relational patterns can be observed in earlier childhood, there may be increased consequences during the adolescent period, when identity is struggling to emerge, and there are also shifts in the most adaptive response to emotion. Parents therefore need to adjust to this developmental phase, shifting in their socialization of emotion and demonstrating flexibility in emotional responding. In infancy and early childhood, focussing on the child’s emotional experience functions to encourage recognition and labeling of emotion supporting basic emotion knowledge and self-regulation (Sanders et al., 2015). By contrast, during adolescence, encouraging excessive focus on emotions, particularly via increased expression of parental reactive emotion or parental matching of emotion, can prolong a negative emotional state resulting in emotion dysregulation and psychopathological risk (Brand and Klimes-Dougan, 2010; Moed et al., 2015). Reassurance or distraction to a positive activity is a more adaptive response better alleviating distress. Yet, magnifying is more commonly used for girls than boys and there is a more robust link between magnifying responses and adolescent psychological problems for girls over boys (Klimes-Dougan et al., 2014). Indeed, girls show higher rates of depression and anxiety than boys, starting in adolescence, and which is associated with internalizing negative emotions of sadness, guilt, and fear (Chaplin, 2015). High expressivity of parental negative emotion relates to internalizing symptoms, depression and anxiety in adolescents (Suveg et al., 2008; Luebbe and Bell, 2014), all of which are associated with development of AN (Adambegan et al., 2012). High levels of parental emotion dysregulation are associated with invalidation of adolescent’s emotional expression, and in turn results in adolescent’s emotion dysregulation (Buckholdt et al., 2014). Equally, by being dismissive of emotion, parents can transmit beliefs that emotions are dangerous or invalid and need to be suppressed (Morris et al., 2007). These beliefs are reported by adults with AN (Hambrook et al., 2011). Therefore, parental emotion socialization is found to be crucial to emotion regulation and adolescent psychological outcomes (Miller-Slough and Dunsmore, 2016), and appears to have a higher cost for female adolescents, and with direct relevance to the development of AN.

Parents enabling the emergence of a child’s own narrative voice is fundamental to development of healthy identity (Fivush and Zaman, 2015). The stories adolescents know about their parents (intergenerational narratives) are critical for understanding self (Fivush and Zaman, 2015). Story telling narratives offer a coherent sense of temporal, autobiographical self, within which discrete experiences can be embedded and understood (Habermas and Bluck, 2000). This is scaffolded by story-telling with parents during early years (McLean and Jennings, 2012). Development of narrative identity is also subject to gender differences. Mothers are especially important in helping their adolescent child to construct narratives around emotion and vulnerability (being overpowered by negative emotion) (McLean and Mansfield, 2012). Mothers employ more emotion words and discuss the causes of emotion when reminiscing with their children than fathers (Fivush et al., 2000). Moreover, both mothers and fathers use more emotion words when constructing narratives with preschool daughters than sons, focusing on elaboration of emotions such as sadness and social-relational themes, with mothers–daughter dyads being most emotionally expressive (Reese and Newcombe, 2007). Meanwhile, adolescent boys receive more supportive scaffolding from mothers than girls do (McLean and Mansfield, 2012). The ability of children and their parents to tell detailed stories about negative emotional past events, cognitive-processing and emotion words, are related to adolescent well-being (Fivush et al., 2008).

It is possible that there is diminished parental scaffolding of emotional narratives and narrative identity is for those who develop AN. It is known that people with AN use fewer words when describing their emotional experiences compared with healthy people or those recovered from AN (Davies et al., 2011, 2012, 2013). People with AN recall over-generalized autobiographical memories, reflecting lack in ability to integrate positive and negative emotional experiences, and which worsens with disorder duration (Nandrino et al., 2006). Memories are associated with less negative emotion expression than healthy people, especially at a lower weight (Brockmeyer et al., 2013). This may reflect poorer narrative identity within which emotions and emotional self cannot be embedded, further impacting difficulties in individuation and self-differentiation.

#### Peers and Identity

During adolescence, emotional and social pressures significantly increase, alongside volume and complexity of social experience. Friendships grow in salience as young people seek out peers for emotional support and explore their identity outside of the family context (Jobe-Shields et al., 2014). Peer interactions become more influential and their quality reflects that of early attachments (Lieberman et al., 1999). Adolescence is therefore considered a time of ‘social reorientation’ (Meeus et al., 2005; Nelson et al., 2016). Influence of peers disproportionately affects behaviors such as risk-taking and relational reasoning during adolescence (Wolf et al., 2015). Adolescents are highly attuned to peer evaluation (Somerville, 2013). They are hypersensitive to social exclusion, particularly in younger adolescence (Sebastian et al., 2010), with older adolescents especially fearful of peer evaluation (Westenberg et al., 2004). There is also acceleration of gender-differentiation and an intensification of gender role expectations (Hill and Lynch, 1983).

Early emotional difficulties outlined above, such as alexithymia, emotion acceptance and regulation, alongside suppression of own emotion and needs, may make an individual particularly vulnerable during adolescence when peer relationships and social acceptance become so vital. Social cognition (mental processes underlying human social behavior and interaction; (Adolphs, 2001) is crucial in successfully negotiating complex social interactions and decisions (Crone, 2013). However, the social brain network undergoes protracted development throughout adolescence before stabilizing in the mid-twenties (Giedd et al., 1999; Gogtay et al., 2004; Sowell et al., 2004; Barnea-Goraly et al., 2005; Shaw et al., 2008). Capacity for introspection or ‘metacognition’ (reflection on our thoughts and behaviors) begins to slowly emerge and gradually improve throughout adolescence (Weil et al., 2013). Younger adolescents are relatively focused on self-oriented choices; impulse control and perspective taking afford increased consideration of consequences for others later in adolescence and early adulthood (Crone, 2013). Further, change in neural and hormonal activity impacts social cognitive abilities, with attachment and mentalization (identifying or inferring mental states of self and others) appearing to enter a state of flux (Jewell et al., 2016). At puberty (around age 11), performance dips on some social cognitive abilities, such as facial emotion recognition and perspective taking, before gradually recovering; a process thought reflect the sudden proliferation of synapses at puberty which are pruned during adolescence (Blakemore and Choudhury, 2006).

People with AN may have impaired emotion recognition and theory of mind (of small to medium effect), that precede AN development, and are further exacerbated by secondary consequences of starvation (Oldershaw et al., 2011; Caglar-Nazali et al., 2014; Bora and Kose, 2016). Difficulties recognizing emotion from blended facial expressions (Dapelo et al., 2016), tone of voice (Kucharska-Pietura et al., 2004; Oldershaw et al., 2010), body movement (Lang et al., 2015) and affective touch (Crucianelli et al., 2016) are reported. A drop in these already potential (trait) difficulties during adolescence is likely to cause distress and increase reliance on external feedback, while simultaneously decreasing ability to define self as a ‘self-in-relation,’ increasing an already impoverished sense of self. Further, identity and other associated self-related processes become a source of information with which to shape decision-making and intrinsic motivations across adolescence (Pfeifer and Berkman, 2018), in the context of strong extrinsic social forces (Wolf et al., 2015). In the absence of a clear developing self, it is possible that extrinsic motivations (e.g., thin ideal; others’ needs) become further reinforced as the key driving force of decisions and behaviors. Thus eating, weight and shape cognitions begin to emerge and drive behavior.

#### Adolescence and Body Image

During adolescence physical shape changes, and awareness of and focus on the body heightens. Adolescents are influenced by and fearful of peer exclusion and evaluation, and this likely includes body and image valuations. EDs are associated with higher family standards on physical appearance (Gunnard et al., 2012). Perfectionism, eating and weight concern are observed at higher levels in mothers of people with AN than in comparison groups (Woodside et al., 2002). The link between insecure (both anxious and avoidant) attachment and EDs is largely mediated by emotion dysregulation, including social comparison (Dias et al., 2011; Ty and Francis, 2013). High externalized self-perceptions and attributions about the importance of weight and shape for popularity and dating by adolescent girls predicts body esteem and eating behavior (Lieberman et al., 2001).

As shape changes, an adolescent vulnerable to AN may receive comments from family or peers that are perceived as external validation. They may seek to manipulate their body through dieting resulting in its conditioned positive reinforcement (Walsh, 2013). Adolescents are particularly sensitive to reward (Steinberg, 2004), with sensitivity to reward and punishment even greater for those at risk of AN (Harrison et al., 2010; Jappe et al., 2011). Initial weight loss is often met with compliments (extrinsic reinforcement); a validation that for a person with the early life experiences described above may finally feel like an achievable goal that is self-motivated (perceived intrinsic achievement).

#### Adolescence: Summary

Adolescence is a time of increased sensitivity to peer rejection and evaluation. It is expected that emotion socialization in infancy and early-mid childhood will afford a child with the foundational skills in emotion recognition, regulation, and expression with which to meet the social and emotional challenges of adolescence. Self-reflection and identity emerging during adolescence is built upon experience and abilities that have been developing since early childhood (Reese et al., 2010). For somebody entering this life stage with poor emotion awareness and a need to please others, such challenges become magnified and may be further exacerbated by familial patterns. The emergence of identity within this context is especially likely to hinge on sources of external evaluation and validation. Thus, poorly integrated identity is built, which, in light of the pervasive ‘thin ideal’ within Western society and increased bodily attention due to puberty, may make somebody particularly drawn to a concrete externally validated sense of self. AN becomes a means to better oneself (Bates, 2015) and to find validity and direction in the face of expectations of what is perceived others want and which is seen as functional and valued.

## Perpetuation of AN: the Ever Decreasing ‘Self’

One of the challenges in working with people with AN is that, once established, there appears an ever-tightening vicious cycle. Increased severity links to increased positive egosyntonic beliefs about the value of AN (Serpell et al., 2004). In many ways, AN becomes self-perpetuating due to its reinforcement of social, emotional and behavioral patterns and the impacts of starvation itself. We argue that these factors further distance an individual from an ‘emotional self’ and thus from recovery; hence AN becomes ‘self-perpetuating’ in and of itself, beyond risk and maintenance factors.

### Emotion Over-Regulation and Perpetuation of Interpersonal Patterns

As discussed throughout the Section “Development of AN,” low levels of expression and high emotion suppression are observed amongst people with AN, including in the context of avoiding conflict (Oldershaw et al., 2015). The impact of emotion suppression in healthy people is increased negative feeling, decreased positive feeling, and decreased emotion expressivity, cardiovascular activity, and oxygenation (Gross and Levenson, 1997; Dan-Glauser and Gross, 2011). Thus, emotion suppression paradoxically leaves somebody with greater negative emotion to regulate, reinforcing their need for emotion regulation strategies. Suppression elicited in experiments with healthy participants reduces interpersonal responsiveness during face-to-face interaction, increasing negative partner-perceptions and hostile behavior (Butler et al., 2007). When watching film clips, people with AN report stimuli incongruent emotions, with limited facial expression (Lang et al., 2016). Inaccurate signaling predicts higher levels of depression and lower levels of well-being, mediated by social connectedness, since accurately signaling emotion states enhances social connectedness (Mauss et al., 2011). Therefore, suppressing emotions, reducing emotion expression and displaying emotions incongruent with those felt, impedes the congruent responding of others. This could perpetuate ‘attunement’ difficulties, echoing those proposed in childhood, further invalidating a true sense of self. Indeed, even when others actively seek to attune to the person with AN, it may paradoxically decrease social connectedness. AN is associated with high levels of internal shame (Grabhorn et al., 2006). People with high levels of internalized shame display shame even following positive task feedback intended to elicit positive emotion (Claesson et al., 2007). Thus, people with AN may experience negative emotions in response to situations more commonly viewed as positive. Further, they have difficulty in distinguishing between positive and negative feedback and report social anhedonia that relates to disorder severity and alexithymia, even following recovery (Wagner et al., 2007; Tchanturia et al., 2012).

There may therefore be numerous ways in which the expected response from others (on both sides) is misinterpreted; social interaction and connection becomes constantly further misattuned. This increases negative emotional experience, particularly shame, and only makes the need for emotional regulation strategies, including social reinforcement, feel more urgent: unhelpful cycles are strengthened, AN becomes embedded and helpful regulation strategies cannot be developed. This fits with reports that people with AN feel they have insufficient emotion regulation strategies available (Harrison et al., 2009; Oldershaw et al., 2015). Actual success in emotion regulation follows expected success (Bigman et al., 2016) further limiting potential emotion regulation abilities, stifling validation of felt emotional experience and cues.

### Starvation Effects

Once AN has developed, starvation effects contribute to self-perpetuation of AN. Starvation reinforces existing psychological difficulties and underpins development of new difficulties. Starvation alone can trigger a reinforcing feedback loop arousing evolved physiological mechanisms, triggering urges to binge eat, increased metabolic efficiency and fat storage, reinforcing fear of fatness and generating renewed attempts at restriction (Nesse, 2017).

#### Interoception

Starvation perpetuates difficulties via its influence on physiological feedback from the body. People with AN struggle to discriminate between bodily sensations (Skǎrderud, 2007), with limited access to physiological experience of emotion when underweight (Miller et al., 2003). Interoceptive cues are abnormally interpreted, resulting in erroneous judgments about internal bodily states (Kaye et al., 2009) and an imbalance between external and internal perception of body relating to ED symptomatology severity (Eshkevari et al., 2014). Thus, there is a disconnection between cognitive and physiological information (Nandrino et al., 2012). There may also be high levels of internal incongruence, with reported emotion mismatching physiological arousal and reactivity (Oldershaw et al., 2011; Nandrino et al., 2012). Low weight further lessens physiological experience of emotion, despite increased self-reported arousal (Miller et al., 2009). This results in interoceptive confusion and the experience of emotions as vague and overwhelming. Such poor interoceptive awareness is increasingly recognized as a core feature of AN, contributing to its emergence and maintenance (Kaye et al., 2009; Nandrino et al., 2012; Strigo et al., 2013).

Interoceptive awareness does not simply inform us of our emotion experience, but of our sense of self; thus disrupted interoceptive processes and feedback, deepened by starvation, further damages emotional sense of self for people with AN. This sense of self is generated, not only by the bottom-up process of interoceptive emotion signals, but also a counter flow of top-down feedback on predictions of their outcomes (such as emotional validation or reflection by others); this establishes accuracy of bottom-up signals and highlights prediction errors (Seth, 2013). Where there is a discrepancy, for example, where what we feel and observe (external feedback) are not highly correlated, we are driven to reduce the discrepancy and revise our predictive model accordingly (Talsma, 2015). This is achieved by altering our sense of internal or external perception. For example, by updating our internal sense of emotional experience to match the one mirrored back to us or updating predictions of the environment in future, with greater reliance on seemingly more precise data (e.g., visual over proprioception/interoception; Seth et al., 2011). It is argued that interoceptive signals in people with EDs are insufficient to accurately predict consequences (Riva and Dakanalis, 2018) or integrate emotional information with sensory experience (Nunn et al., 2008). Even where one does act on one’s felt emotional sense, as described, a lack of attunement may result in non-corresponding feedback. For these reasons, people with AN may be subject to large prediction errors, furthering disruption of interoceptive emotion information and encouraging adjusting or quietening interoceptive feedback (even if felt) in favor of external feedback or heightening desire to manipulate external feedback (e.g., via submissiveness). Inability to integrate or reconcile predictions and confirmations causes persistent anxiety and sense of uncertainty. In this case, minimizing the discrepancy can lead to incorporation of the external signal as part of the self-representation, especially for people with low interoceptive sensitivity (Seth, 2013). Self-objectification (experiencing one’s body via the perspective of an external observer) significantly predicts onset and maintenance of EDs over other more commonly proposed factors (e.g., dieting, body dissatisfaction; Dakanalis et al., 2017).

This hypothesis fits with observed patterns of anxiety, intolerance of uncertainty and over reliance on emotion regulation strategies (via continual checking, worry/rumination processes, submissiveness and social reassurance seeking). It further invalidates internal emotional experience and sense of self. Disrupted interoceptive awareness due to suppression and starvation effects once AN is established may result in an inability to update bodily memory or representation, in part explaining persistent belief in ‘fatness’ or lack of ‘insight’ into the physical severity of the disorder, even when the body becomes dangerously emaciated (Riva and Dakanalis, 2018).

#### The Starved Brain

Abnormalities in brain functioning are reported in AN while ill, and following recovery (Schmidt and Campbell, 2013). Findings of altered brain structure and function demonstrate that the brain is affected by prolonged malnutrition (Bang et al., 2017), increasing damage and functional disability over time (Treasure and Russell, 2011; Treasure et al., 2015a). Indeed, differences in gray matter volume are not observed between newly diagnosed adolescents and healthy comparisons (Olivo et al., 2018). Here, we briefly review key observations in brain functioning for people with AN and how these become exacerbated by the starved state, linking to emotional processing and sense of self.

##### Amygdala

The amygdala is considered the brain’s ‘threat detector,’ central to fear conditioning (Fossati, 2012) and highly responsive to emotional stimuli, particularly faces (Sergerie et al., 2008). Amygdala hyperactivation is observed in adolescents compared with younger children and adults, and does not habituate in anxious individuals due to poorer connectivity with areas providing top-down emotion inhibition (ventromedial prefrontal cortex) (Hare et al., 2008). Adolescents have elevated amygdala-hippocampal complex responses when anticipating social feedback; activation which persists following feedback of rejection for anxious individuals (Lau et al., 2012). Hyperactivity in the amygdala is argued to have a pivotal role in AN (Joos et al., 2011). When exposed to disorder related (e.g., food) and ‘non-disorder related’ stimuli (including emotion stimuli), people with AN exhibit greater amygdala activation (Seeger et al., 2002; Miyake et al., 2010; Joos et al., 2011; Seidel et al., 2018), and heightened fear response (Friederich et al., 2006), indicating heightened emotional arousal (Seidel et al., 2018). Hatch et al. (2010a) argue that for people who develop AN, there is early non-conscious amygdala hypersensitivity to emotion cues, irrespective of weight or nutritional status. It is suggested that bias toward emotional stimuli, poor recognition of facial emotion expression and lack of soothing may be linked to the hyperactive amygdala in AN, persistent following recovery (Oldershaw et al., 2011). Greater ability to tolerate emotion sensations may occur following recovery, in spite of continued increased amygdala activation (Merwin et al., 2013). This suggests a trait amygdala hypersensitivity for people who go on to develop AN contributing to the experience of emotion as overwhelming and aversive, and which may become further heightened during adolescence corresponding with disorder onset.

##### Prefrontal cortex

During displays of negative emotion stimuli, increased activity is also found in the right and left dorsolateral and ventromedial prefrontal cortex (Leppanen et al., 2017; Seidel et al., 2018). Inhibition of negative affect is associated with activation of dorsal anterior cingulate, dorsal medial prefrontal, and lateral prefrontal cortices, and attenuation of brain activity within limbic regions (Phan et al., 2005). This pattern of activity is therefore argued to reflect active control mechanisms in response to emotion, considered particularly necessary in the context of high levels of amygdala activity (Seidel et al., 2018). Studies using resting state and task-based fMRI also appear to support the use of prefrontal mediated cognitive control and self-control processes in AN (Wierenga et al., 2014; Ehrlich et al., 2015; Boehm et al., 2018) with increased prefrontal activation also observed following AN recovery (Wierenga et al., 2014; Ehrlich et al., 2015). Self-control is considered an important mechanism for emotion regulation (Paschke et al., 2016); thus, fits with self-report evidence of emotion over-regulation by people with AN (Oldershaw et al., 2015).

During adolescence, neural adaptations are crucial to the experience of emotion and development of emotion regulation. Self-reported embarrassment and activation in the medial prefrontal cortex are elevated (Somerville et al., 2013). Increased dorsolateral prefrontal activation in girls particularly, coupled with decreased activation in the amygdala, may underpin development of the ability during adolescence to contextualize and regulate emotional experience (Blakemore and Choudhury, 2006). Thus, increased activation of both dorsolateral and amygdala regions for people susceptible to AN (Seidel et al., 2018) may result in a vicious circle of high emotion, poor emotion regulation and need for over-regulation. It is argued that development of orbital and dorsolateral prefrontal cortex regions during and after puberty and increased activity might contribute to excessive worry, perfectionism and strategizing in people with AN (Kaye et al., 2009).

##### Insula

The insula is involved in affective processing and functionally connected with the anterior cingulate cortex, amygdala and ventral tegmental areas (Ham et al., 2013). The insula receives input regarding internal body states (Craig, 2002). It supports visceral, muscular and physiological bodily feedback (Swick et al., 2008) and is involved in anxiety control, regulating disgust, hunger, taste, pain and experience of body image (Suchan et al., 2013). It is implicated in interoception, integrating visual and body perception with emotion and emotional feeling states (Nunn et al., 2008). Access to conscious awareness of interoceptive experience is supported by the posterior insula, while the anterior insula is involved in emotion processing, integration of interoceptive, emotional and cognitive input and contextual integration of interoceptive information (Li et al., 2017). This highlights the role of the insula in emotional awareness and conscious self-representations (Craig, 2002; Seth, 2013).

It is proposed that people with AN are unable to integrate emotional information with sensory experience due to disruptions within the insula (Nunn et al., 2008). Indeed, impaired thalamo-insular circuits are thought to explain a failure of integration of visuospatial information (e.g., pertaining to body image) and homoeostatic (e.g., hunger) signals for people with AN (Geisler et al., 2016). This lack in ability of the insula to integrate basic emotion detection for people with AN occurs in the backdrop of a hyperactive amygdala sending high levels of negative emotional threat information. It is argued that the anterior insula, alongside the amygdala, nucleus accumbens and orbitofrontal cortex are key to supporting our conscious awareness of emotion and integrating this with our sense of self (Seth, 2013). Further, the posterior insula receives and encodes visceral interoceptive input, and increased right posterior insular volume correlates with disorder duration and severity in AN suggesting that difficulties in integrating bottom-up interoceptive information increase with length of disorder (Zucker et al., 2017). Moreover, patterns of insula responding to unpleasant interoceptive states are significantly different for those in remission from AN versus healthy comparison participants (Berner et al., 2018). Altered interoceptive processing within the insula is observed even following recovery from AN (Strigo et al., 2013). It is argued that this may contribute to difficulties predicting and adapting to internal state fluctuation for people with AN, that relates to past AN severity (Berner et al., 2018). These data highlight that disruptions within the insula might link to observations of poor interoceptive awareness for people with AN, and which are exacerbated by disorder duration or severity.

#### Starvation Effects: Summary

People with AN have poor interoceptive awareness exacerbated by low weight. This impedes development and awareness of a sense of self and may be exacerbated by a need to reduce discrepancy between internal and external perception via over-reliance and internalization of external feedback or concrete perceptive cues. People with AN appear to have hyperactive amygdala leading to overwhelming emotional experience and increased prefrontal control mechanisms reducing emotional awareness and clarity. Disruptions within the insula further impair the integration of internal experience with self-awareness and self-representation. This may become more entrenched as the disorder progresses.

### AN as a Lost Sense of Emotional Self

In short, the argument provided outlines AN as a difficulty with attunement and differentiation, leading to a sense of overwhelming emotional experience, that cannot be fully integrated, and is unable to develop into a coherent sense of self during adolescence. This results in poor understanding or perceived value in the individual’s own needs and emotions, resulting in reliance upon external signaling and validation. AN emerges as a means of regulating and managing emotional experience while also providing a false sense of self, including a false sense of needs and a concrete means to meet those needs (e.g., weight and shape goals). Indeed, perhaps the most compelling indication that people with AN lack a known and integrated sense of self is the fact that disorder appears in part to be a quest for this; a “blind search for identity” (Bruch, 1973). The disorder very quickly becomes conflated with actual self, yet at a simplistic and concrete level that can be measurably perfected (“self as weight”; Bates, 2015). The anorexic identity emerges within social interaction and, once established, becomes validated through others’ responses: it achieves basic needs by expressing, communicating and encouraging connectedness from others. In the context of the description of the disorder above, and the self-perpetuation of this cycle, the paradox of AN becomes clear: it is a search for a congruent self, yet one which only removes the sufferer further from an authentic sense of self.

## Clinical Conceptualization and Practice

We have argued that current interventions may be improved if one core hypothesized maintenance factor is isolated and addressed in therapy, with robust assessment of whether the intervention successfully manipulates it. We outlined our argument that in fact several risk and maintenance factors for AN may exert their effects, at least in part, by a single underpinning difficulty with emotion leading to a poorly integrated sense of self. We propose focusing on this putative underpinning factor in a future intervention. This is in keeping with an ‘interventionist-causal model approach’ which allows for independent vetting of a proposed maintenance factor’s relevance, and of the ability of an intervention to manipulate it; achieved by directly assessing whether the intervention on it changes outcomes (Kendler and Campbell, 2009). Using this approach, theoretical models can be successfully translated into practice building a treatment using one hypothesized factor at a time (cf. Freeman et al., 2015). This is consistent with the MRC framework for developing complex evaluations which argues better understanding of expected changes is necessary before embarking on evaluation studies of new complex interventions (Craig et al., 2008).

### Addressing Poor Integrated Sense of ‘Emotional Self’ in Psychological Therapy

In keeping with our hypothesis that a ‘lost sense of emotional self’ forms a core underpinning factor, we argue that working to change this will impact clinical outcomes. Emotion-focussed interventions are supported by empirical evidence and have recently been described as promising for people with AN (Sala et al., 2016). Indeed, since their recognition as putative maintenance factors, interventions have sought to include addressing emotional difficulties (cf. MANTRA, CBT-ED). Yet despite this, as described, limited efficacy above control comparisons remains. In keeping with other authors (Schmidt and Treasure, 2006; Williams et al., 2016), we do not emphasize addressing weight and shape difficulties directly as part of a therapeutic intervention. Indeed, our model is not inconsistent with goals of other treatments, such as developing a ‘non-AN’ identity outlined in MANTRA; yet we explicitly argue the meaning of establishing identity as establishing a core emotional sense of self, which can be flexibly and adaptively used to direct an individual’s needs and relationships. Further, we state that this should be the primary therapeutic goal.

Although emotion is itself inherently complex, maintaining this focus throughout therapy may afford greater opportunity for change. Moreover, we suggest that other interventions do not fully address dfficulties with emotion. It is notable that previous research and intervention development in this area tends to focus upon understanding how emotions arise (e.g., negative appraisals generating guilt or shame), increasing psychoeducation around emotion, and managing difficulties with consequences for emotional experience (i.e., improving regulation). Yet this does not sufficiently address the breadth of difficulties as we view it. Reflecting on her lifetime of work seeking to understand AN, Bruch (1988) describes the therapeutic task: “To help the anorexic patient in her search for autonomy and self-directed identity by evoking awareness of impulses, feelings and needs that originate within herself. The therapeutic focus needs to be on her failure in self-experience, on her defective tools and concepts for organizing and expressing needs, and on her bewilderment when dealing with others” (Bruch, 1988, p. 8). In the 30 years since, there has been considerable effort and attention paid to establishing what the “defective tools and concepts” are and how the “bewilderment when dealing with others” manifests. This has clarified the outward picture of somebody with AN and we have sought to describe herein how this becomes self-perpetuating. As discussed, tackling such factors (e.g., in cognitive-behavioral interventions) may have some benefits; yet we propose that this may become less effective once AN has become established and ‘self-perpetuating.’ We argue that it is the “failure in self experience,” in establishing a felt sense of self through awareness that would naturally lead to self-directed identity, which is missed.

#### Emotion-Focused Therapeutic Approaches

As with all therapeutic approaches, the goals and formulation surrounding the emotions at hand must be carefully considered before applying an intervention or technique. There is a distinction between “primary” emotion as an adaptive inherent “bottom-up” emotional response to a stimulus (Damasio, 2003; Greenberg, 2004; Greenberg and Pascual-Leone, 2006; Barrett et al., 2007) and secondary “top-down” emotions in response to cognitive appraisal (Greenberg and Pascual-Leone, 2006). It has been shown that cognitive reappraisal is effective when used to regulate emotions generated by a “top-down process” (those occurring in response to cognitive appraisal of a situation), but not when applied to those generated by a “bottom-up” process (inherent, often adaptive, emotional response to a stimulus; McRae et al., 2012). Indeed, applying cognitive reappraisal to emotions generated in a “bottom-up” process results in increased amygdala activity, suggesting increased arousal, not regulation (McRae et al., 2012). Furthermore, in low-intensity negative situations, people demonstrate preference for reappraisal, while high-intensity negative emotions are preferably regulated by disengagement distraction, blocking emotional processing before it gathers force (Sheppes et al., 2011). This highlights clear differences in how emotional experience can be most adaptively approached and understood across situations and contexts, dependent upon the emotional need. People with AN may have a persistent disruption in early automatic emotion cues – “primary emotion” (Hatch et al., 2010a). This seems supported by our review findings (Oldershaw et al., 2015) that people with AN report clarity mostly in emotions of guilt and disgust. Such emotions are generally considered to be top-down emotional responses influenced using compassion-focused and cognitive behavioral techniques (Goss and Allan, 2014). While this can improve ED outcomes (Kelly et al., 2014), such treatment may also miss a core feature of AN as described in this article, namely the primary emotional experience.

By the account outlined here, models and interventions for AN should bring focus to core “primary,” “bottom-up” emotional experience. They should seek to embed and integrate this into a self, affording an internal information system of needs to motivate appropriate action, thereby increasing self-efficacy and autonomy. It cannot be that people with AN are not subject to events generating primary emotions. Indeed, as described, hyperactive amygdala suggests a large amount of unconscious early emotion not fully integrated into a felt experience of self; thus undifferentiated, loses clarity and experienced as overwhelming, vague, and negative. Yet, how can one work with something blocked so early that it is experienced only as a vague and confusing state and the continued denial and avoidance of which is highly valued by the experiencer?

Existing psychotherapeutic models may be of benefit when considering how to address this and promote experiential processing of emotion. In Emotion-Focused Therapy (EFT; Greenberg, 2011) the therapeutic process is viewed as co-constructive in which “therapist and client influence each other in non-imposing ways to deepen client experiencing and exploration and promote emotional processing.” (p. 65). The primary goal is to utilize this therapeutic relationship to apply key principles of emotion change (awareness, expression regulation, reflection, transformation and corrective emotional experience). Emotional processing is achieved by recognizing that “the only way out is through” (Pascual-Leone and Greenberg, 2007). Similarly, in Young’s Schema Therapy (ST; Young et al., 2003) a key goal is to be *with* an individual’s primary emotions or ‘core pain’ sometimes described as being ‘housed’ within a ‘vulnerable child mode.’ Time is spent understanding how an individual learnt to cope with their ‘core pain’ through surrendering, avoiding or overcompensating in relation to core maladaptive schema and the costs of these coping styles to their developing sense of ‘self.’

In both EFT and ST, methods of working with emotion are relational and experiential and a core part of therapy relies on chair work (Rice and Greenberg, 1984; Kellogg, 2014). In ST, chair work can map out ‘parts of the self’ and their relationship with core pain or vulnerable child self, with the goal to promote adaptive communication between ‘parts of the self.’ For example, to ‘by pass’ coping modes (or parts of the self) that have learnt to block or deflect primary emotion (such as by ‘cutting off’), such that emotion can be experienced and expressed safely with the therapist and responded to in new and attuned ways. In EFT, parts of the self are most commonly understood as having functions of coach, critic or guard and their relationship with the ‘experiencer’ (which can be adult or child parts of self) are explored to enable primary feelings to emerge and be expressed directly in dialog between the parts, with a process of resolution facilitated (Elliott et al., 2015). One previous adaptation of identifying parts of self to AN is to consider the critical voice specifically as an ‘anorexic voice’ which criticizes using core anorexic cognitions around eating, weight and shape (Dolhanty and Greenberg, 2009; Pugh and Waller, 2017), the power of which relates to disorder severity (Pugh and Waller, 2016).

In both EFT and ST, chair work aims to put parts of the self into live contact, differentiating and intensifying emotion expression and facilitating the identification and expression of an unmet need (Young et al., 2003; Arntz and Jacob, 2012; Elliott et al., 2015). In ST, accessing primary emotion enables the therapist to attune via a re-parenting stance (in a limited way) to some of the unmet needs underneath the core pain (Arntz and Jacob, 2012). In EFT, a distinction is made between the emotional states accessed: those which are adaptive or maladaptive. Once accessed and expressed, primary maladaptive emotions (e.g., core shame; fear of abandonment) can be transformed by putting them in contact with (coactivating) a more adaptive emotional experience (e.g., empowering anger or compassion for the self) to forge a new emotion (Greenberg, 2011) often via expression of needs.

In addition to chair work, further experiential therapeutic tasks and tools enable access to emotional processes and memories. EFT draws upon experiencing-based tasks to respond to in-session client markers, such as experiential focussing to clarify and facilitate feeling shifts (cf. Gendlin, 1981) or systematic evocative unfolding to draw links between stimuli and puzzling reactions to create meaning (Rice and Saperia, 1984). Enactment and imagery can activate adaptive emotional experience for emotion transformation (Greenberg, 2011). ST uses imagery to help access less verbal ‘emotional memories’ for expression and integration into the individuals developing sense of self (Arntz and Jacob, 2012).

While it is beyond the scope of this review to outline these therapeutic models and adaptation to AN in full, we have sought to highlight the compatibility of the EFT and ST models and associated change techniques to working with those with AN, particularly within the context of the conceptualization of a ‘lost emotional self.’ The goal is that a healthy and known self emerges, with a capacity to become aware of, make sense of, regulate, accept, express, and transform emotional experience, thereby using this flexibly and adaptively to navigate themselves, relationships and the world (a reclaimed “conductor”).

## Future Directions in Testing the ‘Lost Emotional Self’ Hypothesis

A clear direction in testing the hypothesis of a ‘lost emotional self’ is to develop a psychological intervention based on the theory, and assess whether: (i) these changes to emotion and self can be achieved, (ii) these changes occur via the proposed mechanisms of enhanced emotional processing, particularly in regard primary ‘bottom up’ emotion, along with less reliance on external cues (and therefore less prediction errors), and an enhanced experience of and belief in core needs and; (iii) this impacts on ED outcomes.

Individual aspects of the model could also be tested. For example, we assert that people with AN experience large perceived prediction errors (Seth, 2013; see the section “Interoception”). This results in integration of external signals as part of self-representation, including ultimately the external perception of their own body as an object. We argue that large prediction errors would predict anxiety, intolerance of uncertainty and reinforce unhelpful emotion regulation strategies. Cross sectional designs could compare those currently ill and those with no illness history, to examine where interoceptive difficulties lie (i.e., objective accuracy, self-evaluated trait interoceptive sensibility or meta-cognitive awareness; Garfinkel et al., 2015). Reduced objective interoceptive sensitivity alongside elevated trait interoceptive sensibility indicates an ‘interoceptive trait prediction error’ (Garfinkel et al., 2016; Seth and Friston, 2016). This could be furthered by comparing with those recovered from AN and examining relationships with anxiety processes and alexithymia.

## Conclusion

We propose AN arises from and perpetuates a lost sense of emotional self; a person without the conductor of the orchestra (our emotional sense of self), persistently reliant upon and sensitive to audience feedback to ascertain if it is performing adequately. This model suggests working to improve awareness, acceptance and valuing of one’s own adaptive interoceptive emotional experience over exteroceptive feedback to achieve emotional validation, emotional self-efficacy and self-agency. Yet this is hampered by high valuing of AN by the sufferer and an increased lack of interoceptive and emotional experience as the disorder progresses, such that AN becomes ‘self-perpetuating,’ creating a ‘stuckness’ in therapy. We propose working in detail with this core putative underpinning maintenance factor and emphasize experiential therapies for psychological engagement with this presentation. This formulation is now to be directly tested, but provides a novel interpretation of existing data, grounded within previous empirical findings.

## Author Contributions

All authors contributed to conception of ideas within the manuscript. AO wrote the manuscript. HS and TL read and gave critical feedback on drafts. All authors approved the final version for submission.

## Conflict of Interest Statement

The authors declare that the research was conducted in the absence of any commercial or financial relationships that could be construed as a potential conflict of interest. The reviewer DV and handling Editor declared their shared affiliation at the time of review.
